# Comparative study of CT-guided radiofrequency and alcohol ablation in the treatment of primary hyperhidrosis

**DOI:** 10.3389/fsurg.2024.1402241

**Published:** 2024-10-28

**Authors:** Yaoping Yu, Jie Cui, Yu Zhang, Lei Feng, Lijun Wang

**Affiliations:** ^1^ Pain Medicine Department, Ningbo Rehabilition Hospital, Ningbo, China; ^2^ Radiology Department, Ningbo Rehabilition Hospital, Ningbo, China

**Keywords:** hyperhidrosis, radiofrequency thermocoagulation, radiofrequency ablation, alcohol ablation, chemical damage, sympathetic ganglion block

## Abstract

**Objective:**

This study compared the efficacy and complications of percutaneous radiofrequency ablation with anhydrous alcohol ablation of sympathetic nerves in treating hyperhidrosis of the head and palms.

**Methods:**

A retrospective analysis was conducted on 54 patients with primary hyperhidrosis in our department from June 2018 to June 2021, divided into a radiofrequency ablation group (30 cases) and an anhydrous alcohol ablation group (24 cases). Treatment outcomes were compared by analyzing the number of CT scans, effectiveness, and complications.

**Results:**

In the radiofrequency group, symptoms of bilateral hyperhidrosis significantly improved in 24 patients, with an 80% postoperative satisfaction rate. In the alcohol ablation group, symptoms significantly improved in 19 patients postoperatively, with a 79.2% satisfaction rate. There was no statistically significant difference in effectiveness or complications between the two groups (all *P* > 0.05). The number of CT scans in the radiofrequency group was 4.60 ± 0.56 and 6.08 ± 0.28 in the alcohol group, showing a statistically significant difference (*P* < 0.05).

**Conclusion:**

This study concluded that both percutaneous radiofrequency ablation and alcohol ablation are effective methods for hyperhidrosis treatment, with similar effectiveness and complication rates, but the radiofrequency ablation group required fewer CT scans.

## 1 Introduction

Primary hyperhidrosis is a chronic disease that does not conform to the body's normal temperature regulation and causes excessive sweating (1, 2). The common sweating sites include heads, armpits, palms, arm sockets, chest backs, and soles. Due to local hyperhidrosis, this disease leads to rubbing erythema, folliculitis, and furuncle. In the winter, the skin of people with hyperhidrosis often lose more moisture due to increased sweating, and result in cracked feet (3–5). In addition, persistent and excessive sweating can seriously affect patients' study, work and social interactions.

There are various treatments for primary hyperhidrosis. First, conservative treatments can be one solution, but they are either not commonly helpful or the effects are usually temporary (6). Second, the main treatment at present is by operations, which is transthoracic endoscopic sympathectomy (TES) (7, 8). Despite of the high success rate, TES also has many limitations. It requires general anesthesia and may bring serious intraoperative problems such as postoperative hematoma, swelling, incision scars and pain (9). Third, the minimally invasive methods of radiofrequency and alcohol ablation sympathetic ganglia are increasingly employed. Many scholars have achieved satisfying results with minimally invasive treatment for hyperhidrosis. This article retrospectively analyzed the comparative study of CT-guided radiofrequency and alcohol ablation to the thoracic sympathetic ganglion for the treatment of primary hyperhidrosis.

## 2 Methods

### 2.1 Patient selection and preoperative and postoperative evaluation

This study collects the treatment information of 54 patients with primary hyperhidrosis of the head and palms from June 2018 to June 2021. There were 29 males and 25 females, aging from 19 to 66 years old. 9 patents only had hyperhidrosis on the head, 10 people had hyperhidrosis on the head and axillary, 18 people had hyperhidrosis on the head and palm, 12 people had hyperhidrosis on the axillary and palm, and 5 people had hyperhidrosis on the palm only. All were in line with the diagnostic criteria for primary hyperhidrosis, referring to the Hyperhidrosis Disease Severity Scale (HDSS Scale) (10).

There were 30 patients in the radiofrequency ablation group and 24 patients in the alcohol ablation group (Table 1).

**Table 1:** Demographic characteristics of two group’ patients (*n* = 54).

	RA group	AA group	*P*
Gender			0.354^a^
Male	18	14	
Female	12	10	
Mean age ± SD, years	29.53 ± 11.38	30.29 ± 13.90	0.831^b^
Mean duration of hyperhidrosis time ± SD, years	8.10 ± 12.66	8.99 ± 5.71	0.392^b^
Distribution of symptoms			
Head only	10	9	0.312^a^
Head and palms	14	10	0.291^a^
Palms only	6	5	0.375^a^

RA, radiofrequency ablation; AA, alcohol ablation.

^a^ *χ*value.

^b^ *t* value.

### 2.2 Surgical procedure

According to the patient's hyperhidrosis distribution, we blocked the T3 or T4 sympathetic ganglion according to the innervation position of the sympathetic ganglion. The surgeries informed patients and their families of the detail of operation process, expected effects and possible complications of CT-guided radiofrequency ablation of thoracic sympathetic ganglion or absolute alcohol blockade therapy, taking T4 ganglion as an example.

**Figure 1:**
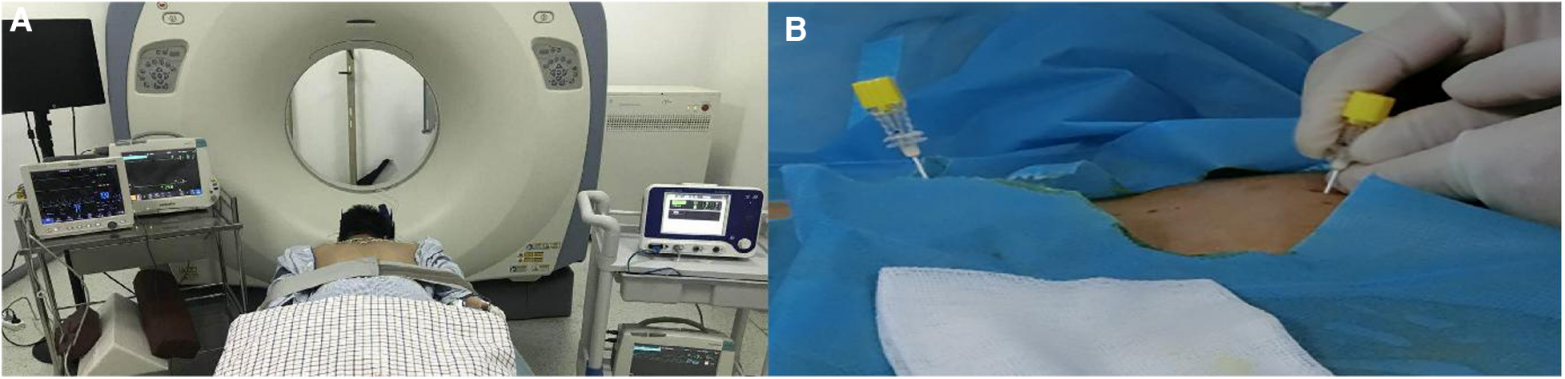
**(A)**: Patient in prone position under CT guidance; **(B)**: The puncture needle is inserted from the chest and back.

**Figure 2:**
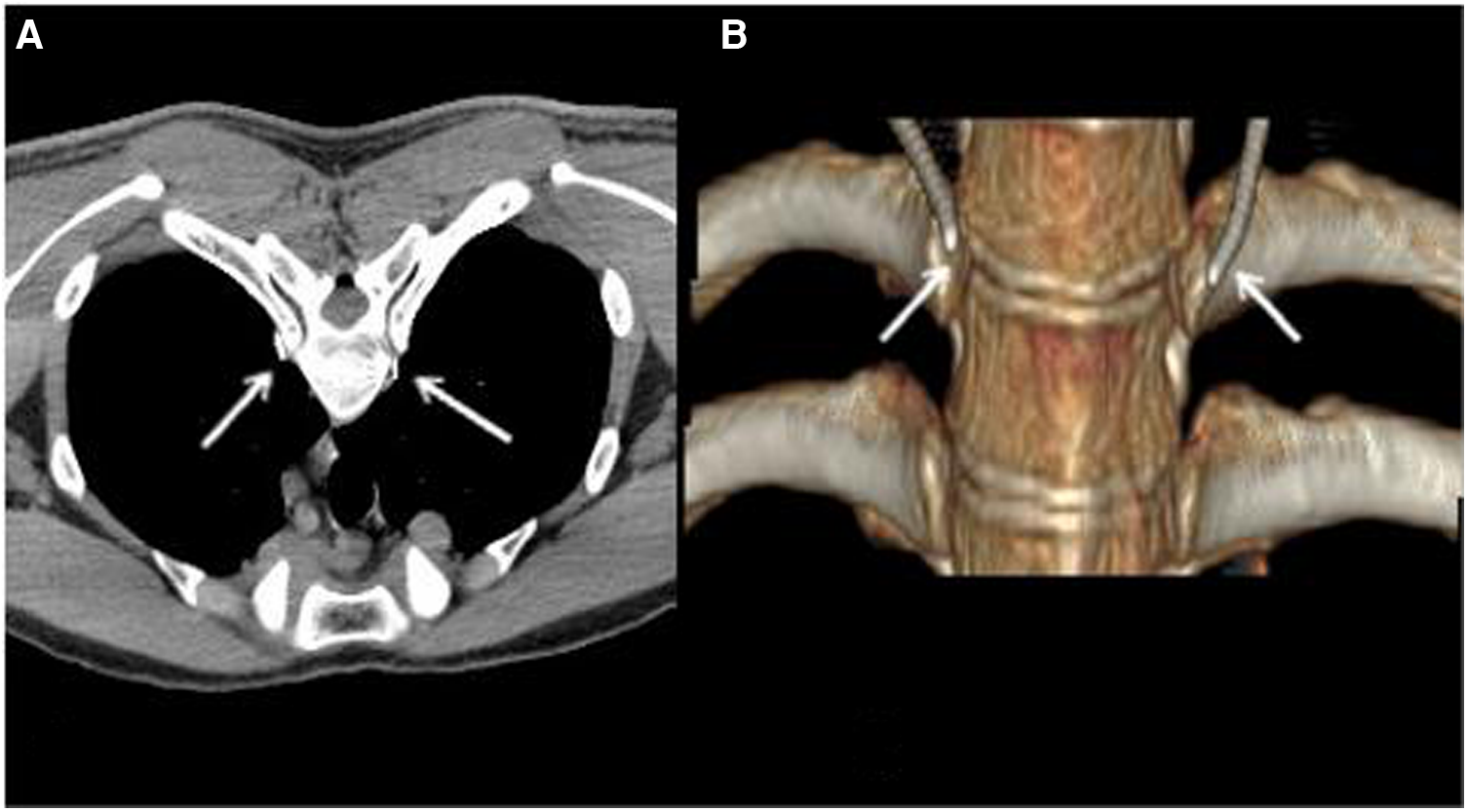
**(A)**: Position after radiofrequency needle puncture; **(B)**: 3D reconstructed image. The white arrow indicated the RF needles.

In the treatment of radiofrequency ablation, we selected the best puncture level and puncture point on the skin after plain scanning, and then planned the needle depth, angle and distance from the needle point to the midline. After the selected puncture point was locally anesthetized, two 7-gauge radio frequency needles were inserted through the T3-T4 paravertebral space on the left and right sides to the upper edge of the posterior lateral T4 rib head of the vertebral body (Figure 1). The position of the needle was tipped to the tip of the test electrode. The position of the radio frequency tip was confirmed by sensory and motor stimulation tests. We then applied thermal stimulation by setting 40 ℃, adjusted the temperature to 75 ℃. The heated coagulation lasts for 90 s and was repeated 3 cycles for radio frequency heat coagulation (Figure 2).

**Figure 3:**
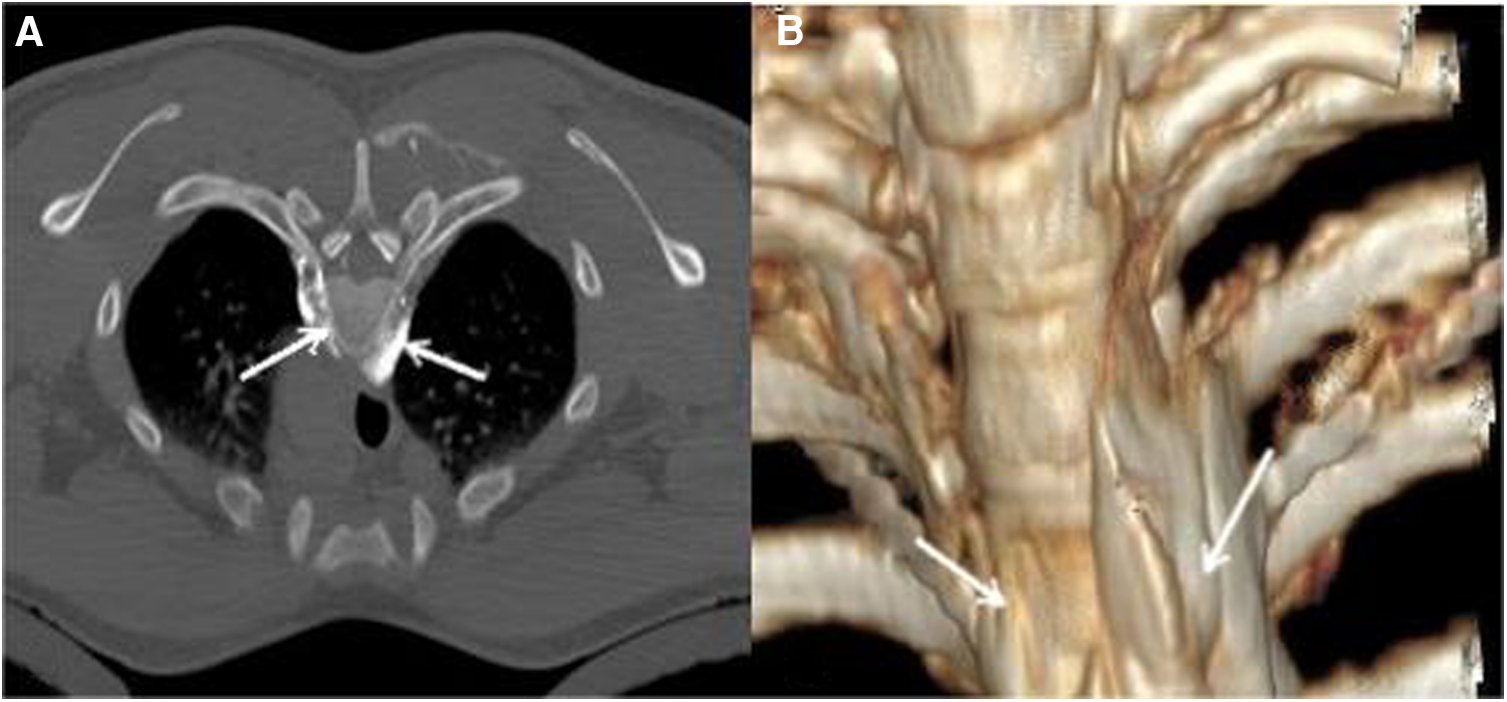
**(A)**: Diffusion of alcohol and contrast medium liquid; **(B)**: 3D reconstructed image. The white arrow indicates the diffusion zone of the mixture of alcohol and iodothyrol.

In the treatment of alcohol, we used a needle to insert the needle through the T3-T4 paravertebral space to the upper edge of the T4 rib head near the vertebral body after positioning puncture. The 1 ml of 30% iohexol injection and 2 ml of 1% lidocaine were injected. CT plain scan showed that the injected liquid was not in the pleural cavity and did not enter the spinal canal. If the diffusion site of the contrast agent was satisfactory, the patient's palms may change from cold to wet and dry after 5 min. Then, we injected 0.5 ml of 30% iohexol injection and 2.5 ml of anhydrous alcohol for chemical blocking. Finally, CT scan showed that the liquid medicine was confined to the vertebral groove between the T3-T4 vertebrae and the wall outside the pleura (Figure 3).

### 2.3 Follow-up and statistical analysis

We followed and evaluated all patients at 1 day, 1, 3, 6, 9, 12, and 18 months after the operation. If the original sweating site reappears in the preoperative state, it refers as the postoperative recurrence. We also collected surgical outcomes, including HDSS score, quality of life (QOL) score for hyperhidrosis (11, 12), postoperative complications, recurrence and the number of intraoperative CT scans. The mean ± standard deviation are reported for continuous variables, and categorical variables were expressed. One-way analysis of variance was used for comparison between groups, and LSD-t test was adopted for further pairwise comparisons with groups. If the *P* < 0.05, it is regarded that the result is statistically significant. The data analysis is conducted by SPSS20.0 statistical software.

## 3 Results

### 3.1 Postoperative curative effect

In the radiofrequency ablation group, the skin of 24 patients who felt hyperhidrosis changed from wet and cold to dry and warm after operation. The HDSS and QLQ scores of patients were significantly improved after the treatment, shown in Tables 2, 3. The average HDSS and QLQ score during the postoperative follow-up period was significantly different from that before the operation (all *P* < 0.05). After the operation, 4 patients had hyperhidrosis in unilateral sites, and 2 patients had hyperhidrosis in bilateral sites. The overall satisfaction rate of the patients reaches to 80%.

**Table 2:** Comparison of HDSS scores of patients before and after treatment of hyperhidrosis in the two groups.

Time	Case		HDSS scores		*P* ^b^
	RA	AA	RA	AA	
Preoperative	30	24	3.63 ± 0.49	3.50 ± 0.51	0.189
1 day	30	24	1.50 ± 0.81^a^	1.50 ± 0.82^a^	0.941
1st month	30	24	1.50 ± 0.80^a^	1.50 ± 0.81^a^	0.941
3th month	30	24	1.50 ± 0.81^a^	1.50 ± 0.82^a^	0.941
6th month	30	24	1.50 ± 0.80^a^	1.50 ± 0.81^a^	0.941
9th month	30	24	1.53 ± 0.80^a^	1.54 ± 0.81^a^	0.917
12th month	30	24	1.56 ± 0.80^a^	1.66 ± 0.85^a^	0.545
18th month	30	24	1.60 ± 0.80^a^	1.67 ± 0.85^a^	0.358

Compared with preoperative, *P^a^* < 0.05 RA, radiofrequency ablation; AA, alcohol ablation, *P^b^:* Compared RA with AA of group in same time.

**Table 3:** Comparison of QLQ scores of patients before and after treatment of hyperhidrosis in the two groups.

Time	Case		QOL scores		*P* ^b^
	RA	AA	RA	AA	
Preoperative	30	24	78.03 ± 10.33	77.41 ± 11.33	0.164
1 day	30	24	34.03 ± 20.42^a^	34.20 ± 20.87^a^	0.898
1st month	30	24	34.33 ± 20.70^a^	34.37 ± 20.86^a^	0.935
3rd month	30	24	34.66 ± 20.99^a^	34.34 ± 20.07^a^	0.821
6th month	30	24	34.80 ± 20.67^a^	35.04 ± 21.36^a^	0.822
9th month	30	24	34.66 ± 20.37^a^	34.83 ± 20.72^a^	0.885
12th month	30	24	34.76 ± 20.59^a^	36.91 ± 21.18^a^	0.589
18th month	30	24	35.50 ± 20.51^a^	37.54 ± 20.99^a^	0.579

Compared with preoperative, *P^a^* < 0.05 RA, radiofrequency ablation; AA, alcohol ablation, *P^b^:* Compared RA with AA group in same time.

In the alcohol group, bilateral hyperhidrosis of 19 patients were significantly improved after operation. The average HDSS and QLQ scores of the patients were significantly improved shown in Tables 2, 3. The difference of HDSS and QLQ scores between postoperative follow-up time and preoperative time was statistically significant (all *P* < 0.05). One patient developed unilateral hyperhidrosis after operation, and the original bilateral hyperhidrosis of 4 patients did not improve significantly. One patient had recurrence after the operation of the 12th month. The patient satisfaction rate was 79.2%. During the same follow-up period, there was no statistically significant difference in the average HDSS and QOL score between the radiofrequency ablation and alcohol groups (*P* > 0.05).

### 3.2 Intraoperative CT scan times

The average number of scan under CT guidance in the radiofrequency ablation group and in the alcohol group was 4.60 ± 0.56 and 6.08 ± 0.28, respectively. The difference between two groups was statistically significant (*P* < 0.05).

### 3.3 Complications

In the radiofrequency ablation group, 5 patients (16.6%) suffered puncture thoracic cavity and lung injury, including 4 with pneumothorax and 1 with hemothorax. No special treatment was given, and they were absorbed by themselves after 1 week. Seven patients (23.3%) developed pain and numbness in the chest, back, armpit o upper arm after operation and were treated symptomatically, which disappeared after 1–2 months. 10 patients (33.3%) developed compensatory hyperhidrosis of the back, and 2 patients (6.6%) developed compensatory hyperhidrosis of the back and feet (Table 4).

**Table 4:** The complications in the two groups.

Surgery group	Effect	Pneumoth-orax (or) hemothor-ax	Pain in the chest, back, armpit, orupper arm	Compensatory hyperhidrosis	Relapse
RA group	24	5	7	12	0
AA group	19	3	3	9	1
*P*	1.00	0.97	0.51	0.41	0.44

RA, radiofrequency ablation; AA, alcohol ablation.

In the alcohol group, 3 patients (12.5%) suffered from pneumothorax during the operation without special treatment and the pneumothorax was absorbed by itself after 1 week. 3 patients (12.5%) developed pain and numbness in the chest, back, armpit or upper arm after operation, and were treated symptomatically, which disappeared after 1–2 months. One case (4.1%) of Horner syndrome was treated without special treatment, and the symptoms improved after one month. 9 patients (37.5%) developed compensatory hyperhidrosis of the back and feet (Table 4). There was no significant difference in complications between the radiofrequency ablation and alcohol blockade groups (Table 4).

## 4 Discussion

Primary hyperhidrosis is component of the disease spectrum of autonomic dysregulation (13). It results from overstimulation of the sweat glands by an inappropriately hyperactive sympathetic system. Studies reporting the prevalence of PH vary it from 1 to 16.3% (14, 15). The disease lowers the life standard, affects psychosocial lives and daily activities, increassthe risk of skin infections. In addition, it is associated with social embarrassment and psychological, professional and emotional problems, significantly reducing quality of life and interfering with regulardaily activities since childhood and aggravated by puberty (16).

There are various treatment methods for primary hyperhidrosis. According to the classification of trauma, it is mainly divided into conservative, minimally invasive, and surgical treatment (17). Conservative treatment methods include sedative drugs, oral anticholinergic drugs, radiation iontophoresis, topical drugs(astringents, antiperspirants, anticholinergic drugs for external use), local injection drugs (botulinum toxin), and psychotherapy (18–20). Drug conservative treatmentpartially mitigate the severity of hyperhidrosis but eventually lose efficacy and are associated with adverse effects (constipation,double vision, xerophthalmia, xerostomia, dizziness).Surgical treatment is mainly transthoracic endoscopic sympathectomy(TES). Although it has a higher success rate, however, it takes general anaesthesia, more complicated operation requirements, relatively expensive surgical costs, higher intraoperative, and postoperative complications (21, 22). That makes doctors seek other less invasive, safe and effective treatments, among which radiofrequency and chemical sympathectomy are increasingly used as minimally invasive techniques (23–25). Our study seems to be the first to specifically retrospectively analyzed and compared the effects and complications of CT-guided radiofrequency and alcohol ablation in treatment of hyperhidrosis in heads and palms.

Wilkinson HA (26) first reported that percutaneous radiofrequency ablation of sympathetic ganglia in 1984. With the assistance of C-arm fluoroscope, they performed radiofrequency thermocoagulation on T2 or T3 for radiofrequency thermocoagulation according to the patients. This report opened the of approach minimally invasive surgery to treat primary hyperhidrosis. Radiofrequency ablation of the sympathetic chain for treatment of PH had been advocated by later scholars (9, 19, 23). The procedure consists in initial localisation of the sympathetic chain, either by fluoroscopy or computed tomography (CT) techniques, followed by percutaneous application of radiofrequency needle (4). The heat of needle was produced by an electric generator and delivered through the tip of a catheter. T2-T4 was the area generally selected to treat PH of palms, heads and armpits. Garcia F et al. (27) used CT-guided radiofrequency thermocoagulation of thoracic sympathetic ganglia to treat hyperhidrosis. They used the QOL score to evaluate patients before and after surgery. According to the scoring standard, the QOL score dropped from 30.3 to 19.8 before surgery. They pointed out that CT guidance could effectively improve the positioning of the ganglion and significantly reduce intraoperative complications.

Dondelinger et al. (28) first proposed CT-guided puncture to chemically block (phenol) the thoracic sympathetic ganglia to treat PH for palms in 1987. Because CT guidance could effectively ensure the safety of chemical liquid diffusion, scholars subsequently used phenol, anhydrous alcohol and other drugs to chemically ablated sympathetic ganglia under CT guidance to achieve better results in the treatment of primary hyperhidrosis (9, 29). So, scholars believed that this method was safe and effective in the treatment of PH, with immediate and long-term effects, and had a lower complication rate than TES of sympathetic ganglia.

Many scholars have used radiofrequency or chemical medicine thoracic sympathetic ganglia to treat hyperhidrosis with a success rate of 62%–85% (16, 27, 29). We achieved satisfying success (80%) in RA group, and the success rate (79.2%) in alcohol ablation group was no obvious difference with RA.

Previous studies had reported that the complications of thoracoscopic sympathetic chain resection mainly include intraoperative hypoxemia, intercostal nerve and blood vessel damage, as well as postoperative lung re-expansion, thoracic adhesions, scar pain, horner syndrome, compensatory hyperhidrosis, and so on (30–32). Among them, although due to the advancement of equipment and the maturity of surgical methods, the incidence of life-threatening or major complications in this operation tended to decrease, but the incidence of compensatory hyperhidrosis could still reach 50 to 90% (33–35). Severe compensatory hyperhidrosis significantly reduced the postoperative quality of life and surgical satisfaction of patients, and it would even turn from complications to primary focal diseases. Purtuloglu et al. (36) conducted a prospective comparative study on the treatment of palm hyperhidrosis by the methods of RA and TES, and found that although the TES was more effective than the RA, however, there was higher and heavier compensatory hyperhidrosis in the TES, so there was no significant difference in patient satisfaction between the two groups.

In our study, the incidence of compensatory hyperhidrosis was 40% in RA group and 37.5% in AA groups, which is relatively lower than previously reported (2, 31). This might be due to selection of lower levels (T3 or T4) for sympathectomy. In addition, the two minimally invasive methods did not completely cut off the thoracic sympathetic chain, and this also conformed to the argument that the sympathetic chain cloud be partly blocked in order to prevent severe compensatory hyperhidrosis by some scholars (31). It was likely that this may explain the no obvious difference in compensatory hyperhidrosis between groups, there seemed to be a positive trend in both groups of patients.

The other complications included the pneumothorax and hemothorax after thoracic cavity and lung puncture injuries, postoperative pain, numbness in the back, underarms or upper arms and horner syndrome. The symptoms disappeared or improved without special treatment. With the aid of CT, the image and positioning during percutaneous puncture were accurate and clear, which made it easier to detect and prevent complications during the operation. Above two minimally invasive treatments of RA and AA, as simple procedure, had a lower complication rates and fewer associate risks than endoscopic electrocoagulation of sympathetic ganglia (6, 9).

In our comparative analysis of CT-assisted radiofrequency and alcohol sympatholysis treatments for heads and palms hyperhidrosis, we find the evidence that the two methods only need local infiltration anesthesia and percutaneous puncture from the back. The treatment effect can reach to a satisfactory rate. There is no obvious difference in satisfaction of two groups. At the same time, there is no significant difference in complications such as chest and lung injuries, chest and back, armpit or upper arm pain and numbness, and compensatory hyperhidrosis. According to the statistics of the number of intraoperative CT scans, the scanning number of anhydrous alcohol groups is slightly more than that of the radiofrequency group. We believe that the main reason is that the former needs to clarify the diffusion position of the contrast fluid under CT-assisted scanning to ensure safety and effectiveness before the final injection of anhydrous alcohol. This increases the number of scans in the alcohol group to some extent.

## 5 Conclusions

This study also has some limitation. The sample size could be increased and the follow-up time could be further extended. Therefore, we look forward to further investigation by using more randomized controlled large-sample studies to further analyze the recurrence rate or other complications of the two treatments. In summary, CT-guided radiofrequency and alcohol ablation sympathicolysis are relatively safe, effective, and minimally invasive treatments with relatively few complications, and can be used as effective options for the treatment of primary hyperhidrosis.

## Data Availability

The raw data supporting the conclusions of this article will be made available by the authors, without undue reservation.
